# Establishment and validation of serum lipid-based nomogram for predicting the risk of prostate cancer

**DOI:** 10.1186/s12894-023-01291-w

**Published:** 2023-07-14

**Authors:** Fu Feng, Yu-Xiang Zhong, Yang Chen, Fu-Xiang Lin, Jian-Hua Huang, Yuan Mai, Peng-Peng Zhao, Wei Wei, Hua-Cai Zhu, Zhan-Ping Xu

**Affiliations:** 1 Department of Urinary Surgery, Foshan Hospital of Traditional Chinese Medicine, No.6, Qinren Road, Foshan, 528099 P.R. China; 2 Guangzhou University of Chinese Medicine, Guangzhou, 510006 P.R. China

**Keywords:** Serum lipid, LDL cholesterol, Triglyceride, Prostate cancer, Nomograms

## Abstract

**Background:**

This study aimed to explore the value of combined serum lipids with clinical symptoms to diagnose prostate cancer (PCa), and to develop and validate a Nomogram and prediction model to better select patients at risk of PCa for prostate biopsy.

**Methods:**

Retrospective analysis of 548 patients who underwent prostate biopsies as a result of high serum prostate-specific antigen (PSA) levels or irregular digital rectal examinations (DRE) was conducted. The enrolled patients were randomly assigned to the training groups (n = 384, 70%) and validation groups (n = 164, 30%). To identify independent variables for PCa, serum lipids (TC, TG, HDL, LDL, apoA-1, and apoB) were taken into account in the multivariable logistic regression analyses of the training group, and established predictive models. After that, we evaluated prediction models with clinical markers using decision curves and the area under the curve (AUC). Based on training group data, a Nomogram was developed to predict PCa.

**Results:**

210 (54.70%) of the patients in the training group were diagnosed with PCa. Multivariate regression analysis showed that total PSA, f/tPSA, PSA density (PSAD), TG, LDL, DRE, and TRUS were independent risk predictors of PCa. A prediction model utilizing a Nomogram was constructed with a cut-off value of 0.502. The training and validation groups achieved area under the curve (AUC) values of 0.846 and 0.814 respectively. According to the decision curve analysis (DCA), the prediction model yielded optimal overall net benefits in both the training and validation groups, which is better than the optimal net benefit of PSA alone. After comparing our developed prediction model with two domestic models and PCPT-RC, we found that our prediction model exhibited significantly superior predictive performance. Furthermore, in comparison with clinical indicators, our Nomogram’s ability to predict prostate cancer showed good estimation, suggesting its potential as a reliable tool for prognostication.

**Conclusions:**

The prediction model and Nomogram, which utilize both blood lipid levels and clinical signs, demonstrated improved accuracy in predicting the risk of prostate cancer, and consequently can guide the selection of appropriate diagnostic strategies for each patient in a more personalized manner.

**Supplementary Information:**

The online version contains supplementary material available at 10.1186/s12894-023-01291-w.

## Introduction

Prostate cancer (PCa) remains the most common cause of cancer-related mortality among men worldwide, and they were rapidly increasing in Asia as well [1]. Westernization of lifestyle, especially the adoption of high-fat diets, resulted in an increased incidence of PCa in countries that previously had the lowest risk [2], making early prostate cancer screening more crucial. Indeed, several types of diets had been linked to advanced PCa, hypercholesterolemia has also been linked to aggressive PCa [3].

In clinical practice, prostate biopsy has long been recognized as the most reliable method for identifying and diagnosing prostate cancer, earning its status as the gold standard. The decision to perform a prostate biopsy is influenced by various factors, including the level of serum prostate-specific antigen (PSA) and its related factors, as well as the findings of a digital rectal examination (DRE) and imaging. However, the commonly employed methods for PCa screening, such as the PSA test, were subjective but not specific enough. It led to the increased risk of unnecessary invasive prostate biopsy. Then, alternate PCa diagnosis strategies were required to prevent men who are unlikely to have prostate cancer from undergoing potentially morbid or invasive procedures.

To address these issues, clinical prediction models had been developed in some Western countries to help inform medical decisions by assessing the risk of cancer in a population individually, thereby reducing unnecessary punctures and increasing positive puncture rates, which were more accurate and scientific than traditional methods based on PSA and DRE. So far, more than a dozen models have been developed in Western countries to predict prostate cancer risk, with the PCPT-RC [4] being the most widely validated and promoted. Recently, several domestic researchers have developed clinical prediction models that incorporate multiple clinical parameters in the Chinese population, such as Wu et al. (domestic model 1) [5] and Tang et al. (domestic model 2) [6]. However, the clinical variables included in these predictive models need to be further explored and fail to further consider the effect of a high-fat diet on prostate cancer.

Cholesterol was an essential component in the plasma membrane [7]. Lipid rafts are cholesterol and sphingolipids-enriched membrane microdomains within the lipid bilayer which modulates cell signaling [8]. Increasing evidence suggested that cholesterol might contribute to the development, aggressiveness, and progression of prostate cancer via its effects on inflammation and steroidogenesis [9]. Additionally, the level of cholesterol was found to be associated with PSA levels [10]. It was also considered to contribute as a substrate for the creation of intra-tumoral androgen at all phases of disease [11, 12], including metastatic castration-resistant prostate cancer (mCRPC) [13]. In addition, an in vivo model in mice fed a high-cholesterol diet demonstrated a higher risk of tumor development and progression [14–16], whereas cancer progression models in vitro demonstrated anomalies in cholesterol metabolism regulators [17]. Furthermore, accumulating data suggested that cholesterol-lowering statins were associated with a lower risk of advanced and fatal prostate cancer [18–20].

Thus, an analysis of serum lipid profiles, such as TC (cholesterol), TG (triglyceride), HDL (high-density lipoprotein), LDL (low-density lipoprotein), apolipoprotein AI (apo AI), apolipoprotein B (apo B), and blood PSA levels was conducted retrospectively to determine if serum lipid profile may help optimize prostate cancer diagnosis.

## Materials and methods

### Recruiting patients

From January 2016 to December 2022, 800 patients who underwent transrectal prostate puncture guided by transrectal ultrasonography (TRUS) (12 + X core, all patients underwent preoperative magnetic resonance imaging (MRI) of the prostate, followed by 12-core systematic biopsy with or without targeted biopsy based on the MRI results) in the department of urinary surgery of Foshan Hospital of Traditional Chinese Medicine were recruited. All prostate biopsies were conducted by urologists with more than ten years of working experience in our department. The indications for prostate biopsy were the following: (1) tPSA > 10.0 ng/ml. (2) tPSA > 4.0ng/ml and < 10.0ng/ml with suspicious fPSA/tPSA < 0.16. (3) Affirmative outcomes identified through a DRE for any level of tPSA, and (4) positive findings from imaging techniques like TRUS and MRI have been observed regardless of the level of tPSA. The exclusion criteria were as detailed below: (1) 20 patients previously treated with prostate surgery or previously diagnosed with prostate cancer. (2) 43 patients who received 5 alpha-reductase inhibitors or who had urethral catheters in place, or who had previously undergone invasive therapy for benign prostatic hyperplasia (BPH). (3) 32 patients with acute bacterial prostatitis or other inflammatory systemic illnesses. (4) 54 patients without complete clinical data. (5) 103 patients with high blood pressure, diabetes, or taking lipid-lowering drugs, these patients may have taken lipid-lowering medication in the past or currently. 548 cases were included eventually. The study received official approval from the ethics committee of Foshan Hospital of Traditional Chinese Medicine (20220520). Before transrectal ultrasound-guided prostate puncture, all patients provided informed consent.

### Clinical information

The baseline information was retrospectively acquired from the clinical digital information including age, BMI (body mass index), serum PSA (total PSA and free PSA), f/tPSA (free/total PSA ratio), prostate volume (PV), prostate-specific antigen density (PSAD), TC, TG, HDL, LDL, apo A-1, apo B, DRE results, TRUS finding. f/tPSA was calculated using free PSA and total PSA. PV was based on TRUS, calculated as 0.52 × length × width × height. PSAD was computed by combining total PSA and PV.

### Nomogram development and evaluating

To construct an accurate nomogram that predicts the likelihood of PCa, we carried out multivariable logistic regression analyses to determine the correlation between the relevant factors and PCa. We utilized the forward method in multivariable logistic regression analysis to identify independent predictors, and subsequently, to determine the significant variables for creating nomograms. To assess the risk model’s utility, receiver operator characteristic (ROC) curves were generated and the corresponding area under the curves (AUC) values was compared. To determine the statistical significance between the AUC values, the DeLong method was employed. The Nomogram was developed by randomly selecting 384 (70%) cases, with 164 (30%) cases set aside for validation by the R package “verification”. A nomogram that predicts the risk of PCa was developed based on independent risk factors. The calibration curve was employed to evaluate the consistency of nomogram-predicted probabilities with observed probabilities. To evaluate the net benefit obtained from utilizing the developed nomogram, a decision curve analysis (DCA) was performed. Data were plotted using R software v.4.1.0 (R Foundation, Vienna, Austria) for the nomogram (R package ‘rms’), calibration plot (R package ‘rms’), and DCA (R package ‘rmda’).

### Statistical analysis

The distribution of categorical variables was evaluated using Pearson’s Chi-square test, while the distribution of continuous variables was assessed using either the Mann-Whitney U test or Student’s t-test, depending on the distribution of the data. The multivariable logistic regression analysis was conducted using the SPSS 24.0 software. (SPSS Inc., Chicago, IL). The AUC, sensitivity, specificity, and positive and negative predictive values (PPV/NPV) for each method were calculated using MedCalc 19.3. The optimal cutoff values for the biopsy threshold, which balanced sensitivity and specificity, were determined using Youden’s J index (sensitivity + specificity − 1). Statistical significance was considered for all tests with a two-sided p-value less than 0.05.

## Results

### Baseline clinical characteristics

**Fig. 1:**
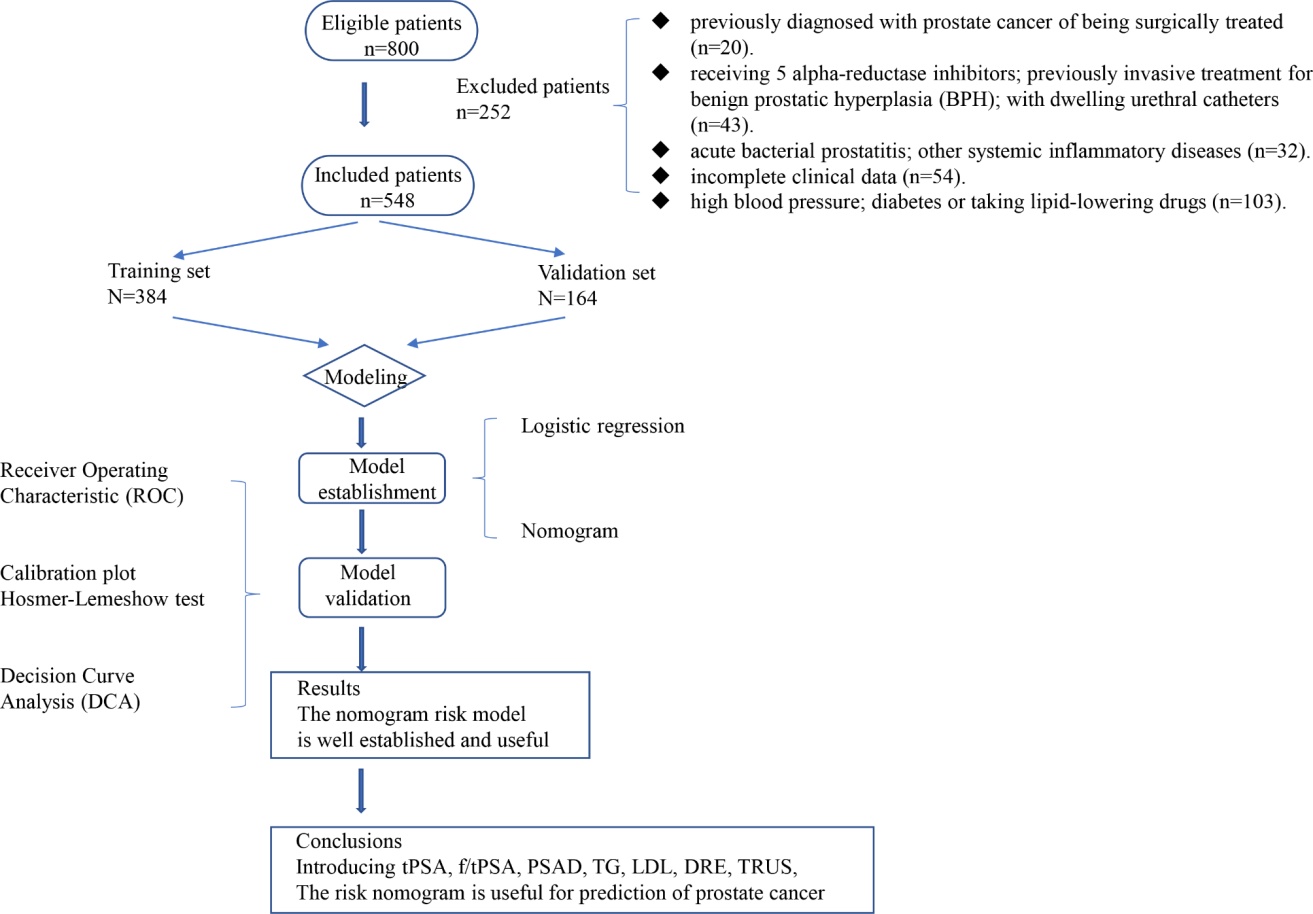
Flow diagram of study design. tPSA, free prostate-specific antigen; TG, Triglyceride; LDL, Low-density lipoprotein; DRE, digital rectal exam; TRUS, Transrectal Ultrasonography;

In this study, a total of 548 patients who satisfied the inclusion criteria were included. Figure 1 was a flowchart describing the selection of patients and data from the hospital information system. 384 (70%) and 164 (30%) men were in the training and validation groups, respectively. As compared to baseline data, there was no discernible difference in the general condition of patients between the training and validation groups (Table 1), characters of age, BMI, tPSA, f/tPSA, TC, TG, and other indicators(P > 0.05).

**Table 1:** Patient demographics and clinical characteristics of the Training group and Validation group

Demographic characteristics	Total	Training group		Validation group		P-value
		No cancer, n(%)	PCa, n(%)	No cancer, n(%)	PCa, n(%)	
**Age (years)**						0.394
< 60	70	18	28	11	13	
≥ 60	478	192	146	76	64	
**BMI (kg/m** ^**2**^ **)**						0.53
< 18.5	25	5	10	5	5	
[18.5, 23.9)	193	79	57	29	28	
≥ 23.9	330	126	107	53	44	
**PSA (ng/ml)**						0.292
< 4	246	132	46	53	15	
≥ 4	302	78	128	34	62	
**f/t PSA**						0.111
< 0.16	239	68	91	30	50	
≥ 0.16	309	142	83	57	27	
**PSAD**						0.466
< 0.15	307	143	76	59	29	
≥ 0.15	241	67	98	28	48	
**TC (mmol/L)**						0.278
< 5.69	471	177	149	74	71	
≥ 5.69	77	33	25	13	6	
**TG (mmol/L)**						0.843
< 1.7	424	175	123	72	54	
≥ 1.7	124	35	51	15	23	
**HDL (mmol/L)**						0.53
< 1.55	485	194	148	80	63	
≥ 1.55	63	16	26	7	14	
**LDL (mmol/L)**						0.805
< 3.37	424	181	115	69	59	
≥ 3.37	124	29	59	18	18	
**apoA-1 (g/L)**						0.501
< 1.7	526	206	164	83	73	
≥ 1.7	22	4	10	4	4	
**apoB (g/L)**						0.815
< 1.55	228	93	68	35	32	
≥ 1.55	320	117	106	52	45	
**DRE**						0.155
Negative	367	149	101	73	44	
Positive	181	61	73	14	33	
**TRUS**						0.69
Negative	339	152	95	52	40	
Positive	209	58	79	35	37	

**BMI**, Body Mass Index; **PSA**, Prostate specific antigen; **TC**, total cholesterol; **TG**, Triglyceride; **HDL**, high-density lipoprotein; **LDL**, Low-density lipoprotein; **apoA-1**, apoprotein A-1; **apoB**, apoprotein B; **DRE**, digital rectal exam; **TRUS**, Transrectal Ultrasonography;

### Multivariable analysis predicting PCa

**Fig. 2:**
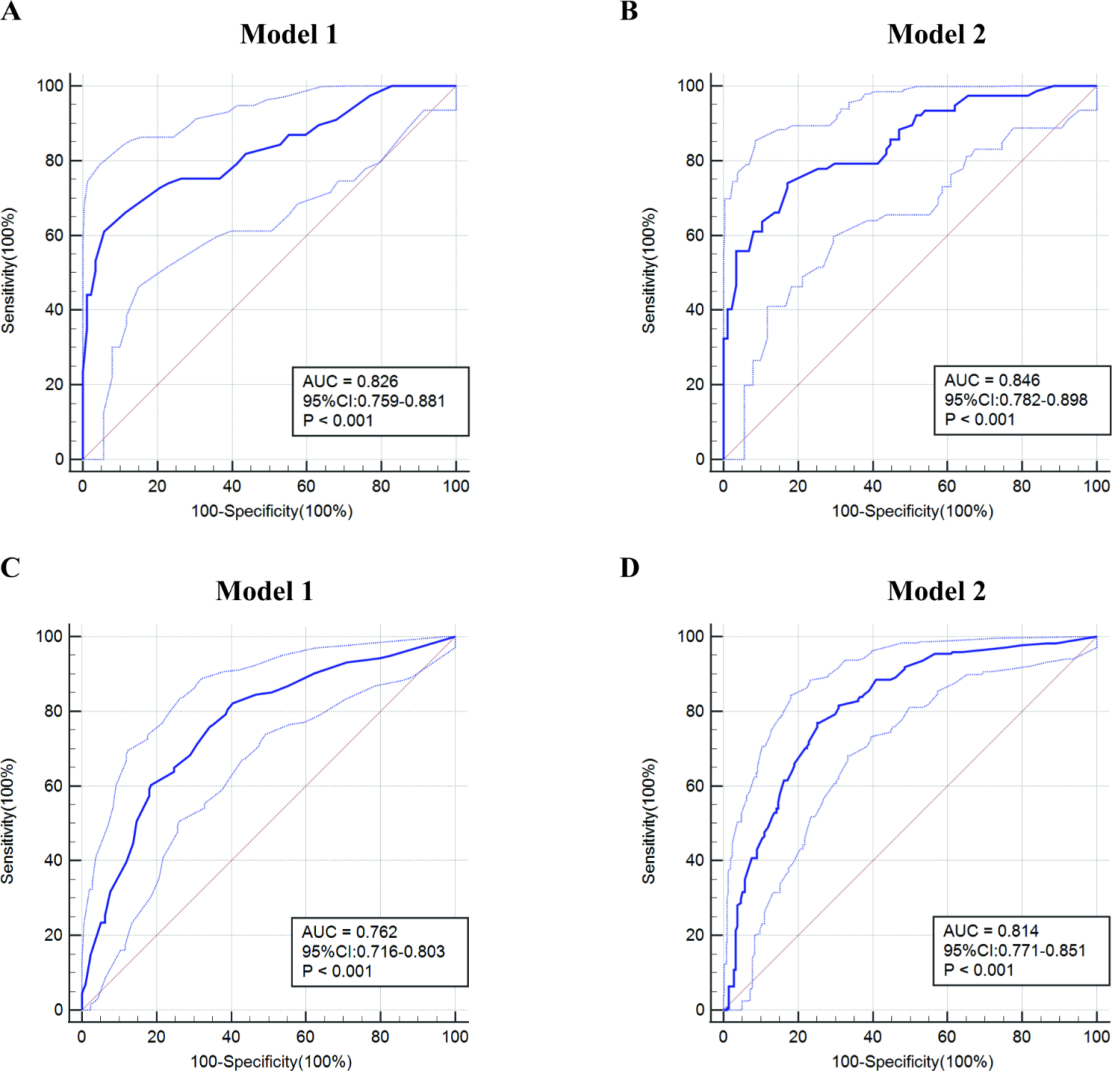
Receiver operating characteristic curve (ROC) of Model 1 and Model 2 for predicting prostate cancer risk. The *y*-axis represents the true positive rate of the risk prediction, the *x*-axis represents the false positive rate of the risk prediction. The thick blue line represents the performance of the predictive model and the light blue dotted line represents the 95% CI in the training set of Model 1(**A**) and Model 2(**B**) and the validation set of Model 1(**C**) and Model 2(**D**)

In multivariable logistic regression analysis, tPSA (P < 0.001), f/tPSA (P = 0.018), PSAD (P = 0.030), TG (P = 0.006), LDL (P < 0.001), DRE (P = 0.002) and TRUS (P = 0.009) were significantly related to the presence of PCa in biopsy (Table 2). Ultimately, two prediction models were established. Model 1 integrated common clinical indicators, including tPSA, f/tPSA, PSAD, DRE, and TRUS (Additional File Table 1); the common clinical indicators (tPSA, f/tPSA, PSAD, DRE, TRUS), TG, and LDL were incorporated in model 2 for detection of PCa to compare whether the inclusion of blood lipids (TG and LDL) allows further optimization of the prediction model (Additional File Table 2). In terms of predicting PCa detection rates, model 1 and model 2 demonstrated AUCs of 0.826(0.759–0.881, P < 0.001) and 0.846(0.787–0.905, P < 0.001) for the training group and 0.762 (0.716–0.803, P < 0.001) and 0.814 (0.771–0.857, P < 0.001) for the validation group respectively (Fig. 2). According to the ROC curve analysis, Model 2 demonstrated a significant superiority over both Model 1 and PSA in both the training and validation group (Table 3).

**Table 2:** Multivariate logistic regression models in the training group

Parameters	Multivariate model		
	OR	95%CI	P
**Age (years)**	0.571	0.258–1.217	0.147
**BMI (kg/m** ^**2**^ **)**	0.504	0.149–1.709	0.272
**t PSA (ng/ml)**	4.336	2.551–7.370	**< 0.001**
**f/t PSA**	1.826	1.107–3.012	**0.018**
**PSAD**	1.732	1.054–2.849	**0.030**
**TC (mmol/L)**	0.918	0.444–1.896	0.817
**TG (mmol/L)**	2.462	1.299–4.667	**0.006**
**HDL (mmol/L)**	1.999	0.740–5.395	0.172
**LDL (mmol/L)**	4.680	2.419–9.055	**< 0.001**
**apoA-1 (g/L)**	0.552	0.119–2.573	0.450
**apoB (g/L)**	0.717	0.411–1.250	0.241
**DRE**	2.380	1.380–4.105	**0.002**
**TRUS**	2.054	1.200-3.515	**0.009**

**Table 3:** Diagnostic accuracy of our model and other models using validation group

Prediction model	AUC	95% CI	*P*-value (AUC)
**Our Model 2**	0.814	0.771–0.851	< 0.001
**Our Model 1**	0.762	0.716–0.803	< 0.001
**Domestic Model 1**	0.732	0.684–0.775	< 0.001
**Domestic Model 2**	0.729	0.682–0.773	< 0.001
**PCPT-RC Model**	0.728	0.680–0.772	< 0.001
**PSA**	0.682	0.633–0.728	< 0.001

### Nomogram to estimate the risk of PCa

**Fig. 3:**
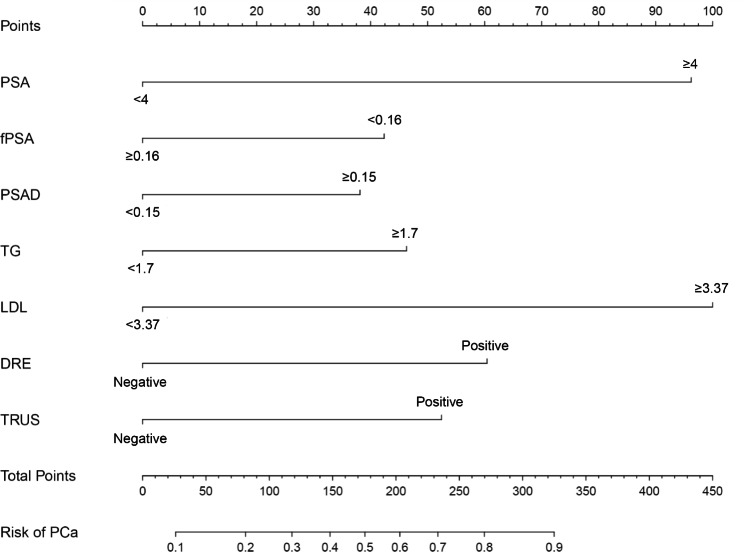
Nomogram predicts the probability of PCa.

Using multivariate logistic regression, a predictive model was constructed. 7 of the initial 13 variables were incorporated into the predictive model (model 2), namely tPSA, f/tPSA, PSAD, TG, LDL, DRE, and TRUS, as predictors. Each variable is scored, and a straight line is drawn from the total score to determine harboring PCa probability (Fig. 3). When the Youden index reached its peak value, the cut-off value for prediction model 2 was determined as 0.502 in the training group. The evaluation metrics of sensitivity, specificity, positive predictive value, negative predictive value, false negative rate, and false positive rate were 74.03%, 82.76%, 78.3%, 25.97%, and 17.24%, respectively (Table 4). Our developed forecasting model was further validated using a validation set of 164 cases in combination with PCPT-CRC and two other domestic forecasting models. The AUC values of our prediction model 2, domestic model 1, domestic model 2, and PCPT-CRC were 0.814, 0.732, 0.729, and 0.728, respectively (Table 3). Comparing our prediction model with two domestic prediction models as well as PCPT-CRC, statistically significant differences were found (P < 0.001, Table 5).

**Table 4:** Diagnostic values of models in the training group and validation group for the results of prostate biopsy

Prediction model	Cutoff	Youden index	SEN (%)	SPE (%)	PPV (%)	NPV (%)	FNR (%)	FPR (%)	AUC	P-value (AUC)
**Model 1(Training group)**	> 0.641	0.553	61.04	95.25	90.4	73.2	38.96	4.75	0.826	< 0.001
**Model 2(Training group)**	> 0.502	0.568	74.03	82.76	79.2	78.3	25.97	17.24	0.846	< 0.001
**Model 1(Validation group)**	> 0.519	0.418	60.34	81.43	72.9	71.2	39.66	18.57	0.762	< 0.001
**Model 2 (Validation group)**	> 0.440	0.518	77.01	74.76	71.7	79.7	22.99	25.24	0.814	< 0.001

**Table 5:** Comparison of diagnostic values of other prediction models with that of our models

Prediction model	AUC	*P*-value
**Our Model 2**	0.814	N/A
**Our Model 1**	0.762	< 0.001
**Domestic Model 1**	0.732	< 0.001
**Domestic Model 2**	0.729	< 0.001
**PCPT-RC Model**	0.728	< 0.001
**PSA**	0.682	< 0.001

### Predictive model validation

**Fig. 4:**
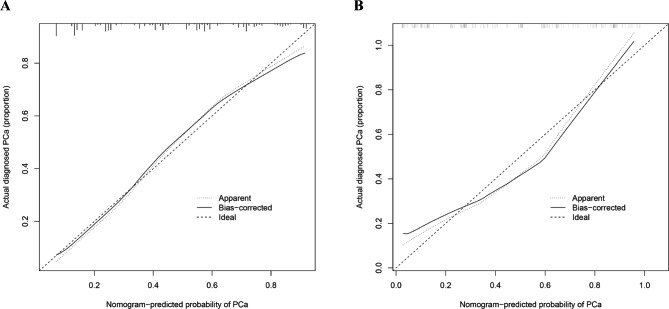
Calibration curves of the predictive prostate cancer risk nomogram. The *y*-axis represents actual diagnosed cases of prostate cancer, the *x*-axis represents the predicted risk of prostate cancer. The diagonal dotted line represents a perfect prediction by an ideal model, and the solid line represents the performance of the training set (**A**) and validation set (**B**), with the results indicating that a closer fit to the diagonal dotted line represents a better prediction

**Fig. 5:**
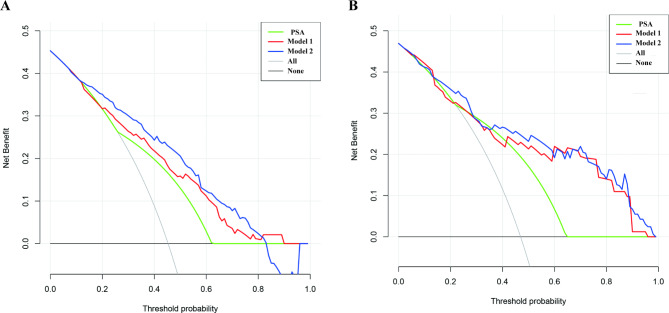
Decision curve analysis for the prostate cancer risk nomogram. The *y*-axis measures the net benefit. The thick solid line = net benefit when all patients have no prostate cancer, the thin solid line = net benefit when all patients have prostate cancer, the solid green line = PSA, the solid red line = Model 1, solid blue line = Model 2. **A** from the training set, **B** from the validation set

The calibration plot, which used internal bootstrap sampling (n = 1000), indicated a high degree of consistency between the bias-corrected curve and the ideal curve (the 45-degree line) both in the training and validation sets, indicating that our nomogram accurately predicted the occurrence risk and demonstrated good calibration (Fig. 4). To determine whether the two models and clinical indicators had potential clinical benefits, we conducted DCA in the training and validation groups. We evaluated the overall utility of the decision models, including PSA, model 1, and model 2. When the threshold probability was 0.06–0.82, the net benefit of the two models was higher than that of PSA. Model 2 demonstrated the greatest net benefit, outperforming all other indices (Fig. 5).

## Discussion

PCa is a complicated disease characterized by phenotypes, and each patient appears to have an “individual” process. The lifestyle and nutritional preferences of an individual could influence the development of PCa. There was strong evidence that obesity, hyperlipidemia, and other components of metabolic syndrome significantly elevate the risk of developing prostate cancer [21]. In this population-based retrospective study, we aimed to evaluate the correlation between blood lipid levels and the risk of prostate cancer. We found that the increased risks of prostate cancer were associated with high levels of both serum TG and LDL cholesterol.

Although the relationship between dyslipidemia and PCa has been extensively explored, the underlying mechanism remains unclear. Many hypotheses have been offered to explain the association between lipids and the growth and development of PCa. Activation of the sterol regulatory element-binding protein pathway by androgens promoted the expression of lipogenic genes, which stimulated the accumulation of neutral lipids (triglycerides and cholesteryl esters) and tumorigenesis, thereby stimulating prostate tumor growth [22]. Increasing lipogenesis was expected to contribute to prostate cancer progression [23]. Furthermore, animal studies had demonstrated that LNCaP tumors, which are human prostate cancer cells sensitive to androgens, grow more slowly in nude mice on a low-fat diet with low serum triglyceride levels [24].

Multiple studies indicated that elevated LDL levels were associated with an increased risk of cancer. LDL promoted the proliferation, migration, and invasion of prostate cancer cells by inducing JAK1/JAK2/STAT3 activation and upregulated the expression of several oncogenic gene products [3]. Binding of LDL to the LDL receptors (LDLR) induced transcription of sterol regulatory element binding proteins (SREBPs) through activation of the PI3K/Akt/mTOR signaling pathway, thereby promoting cholesterol uptake and synthesis for cancer cell development and progression [25]. In addition, some researchers have shown that highly metastatic tumor tissue contained high levels of LDL and formed more oxidized low-density lipoprotein (ox-LDL), which induced neutrophil migration and accumulation via the ox-LDL axis to create a highly metastatic tumor microenvironment [26].

In contrast to our findings, some studies discovered that lower LDL levels in RP patients were an independent predictor of prostate cancer recurrence [27]. The suggested explanation was that cholesterol was crucial for the metabolism of prostate cancer, and the decreased LDL levels may represent the aggressive character of prostate cancer. Nonetheless, they were unable to determine whether decreased LDL levels were a modifiable risk factor or a consequence of tumor metabolism.

According to our investigation into the relationship between TG and prostate malignancies, we found that elevated levels of serum TG were associated with a higher risk of developing prostate cancer. The metabolism of TG produces vital fatty acids [28]. It had been proposed that when LDL serum level was high, the loss of control of the LDL receptor allows for increased intake of essential fatty acids in prostate cancer. It promotes the synthesis of prostaglandin E2 (PGE2), which was a well-known growth factor for cancer cells [28, 29]. TG provide to the necessary fatty acids available for PGE2 synthesis, which could explain the link between TG and the risk of prostate cancer. However, epidemiological research on the link between serum TG and prostate cancer risk had shown mixed results. Ulmer et al.[30] demonstrated a negative correlation between TG and the risk of prostate cancer, although Wuermli et al.[28] detected a positive correlation and Lund et al.[31] found no significant correlation. Our findings were consistent with Wuermli et al. [28], that elevated serum triglyceride level was positively associated with a higher incidence of prostate cancer.

Additionally, hypertriglyceridemia was characterized by the presence of small dense LDL particles and cholesterol-rich remnant lipoproteins (RLP), which were the products of the hydrolysis of chylomicrons and VLDL [32, 33]. It was through the ApoE and ApoB/E receptors that RLPs bonded to cells. According to in vitro experiments, 1 g/mL of RLP stimulated PC-3 prostate cancer cell growth in a dose-dependent way. This dose was similar to those detected in patients with 10 ng/dL [34]. These findings should be investigated further in an in vivo model.

Lipids were routinely checked by clinical patients, and this was a simple procedure that patients readily accept. We found that the ROC curve study revealed that the prediction model 2 was significantly superior to model 1, PCPT-CRC, and two domestic prediction models, suggesting that lipids plus the common clinical indicators (tPSA, f/tPSA, PSAD, DRE, TRUS) were much more predictive of prostate cancer patients than the common clinical indicators alone, which not only improved the biopsy rate of patients with prostate cancer but also reduced the need for unnecessary invasive testing. In consideration of the potential risks associated with false-negative prostate cancer and complications of prostate biopsy, we determined a cut-off value for AUC when the Jorden Index reached its optimal level. The cut-off value of AUC in the training group was determined to be 0.502. The sensitivity, specificity, positive predictive value, negative predictive value, false negative rate, and false positive rate were determined to be 74.03%, 82.76%, 78.3%, 25.97%, and 17.24%, respectively. Through the incorporation of a threshold value of 0.502 in the training group, our prediction model successfully prevented 95.25% of unnecessary prostate biopsies with 38.96% of missed positive cases. Based on our findings, it is recommended to perform a prostate biopsy when the prediction probability exceeds 0.502, while active monitoring may be considered for cases below this threshold.

Despite these promising results, our study is not without limitations: (1) this study was retrospective, hence, selection bias was inevitable; (2) The sample size of the single-center study was modest, and a subsequent multicenter cooperative study was required to confirm the conclusion; (3) The prediction model lacked external validation and follow-up external multicenter data were required; (4) A risk calculator developed and validated in this study was only used to predict prostate cancer on initial prostate biopsy, but failed to distinguish between high-grade and low-grade prostate cancer. In our further studies, we would work in predicting the model of high-grade prostate cancer (defined as a Gleason score sum of 7 or higher).

In summary, the main objective of our study was to evaluate the diagnostic efficacy of combining blood lipid levels and clinical symptoms in the diagnosis of prostate cancer. We established an individualized nomograph prediction model to help identify patients with prostate cancer at an early stage and provide individualized risk calculation to reduce unnecessary puncture biopsies.

## Electronic supplementary material

Below is the link to the electronic supplementary material.

Additional File Table 1: Multivariate stepwise logistic regression analysis for predicting PCa in the training group for Model 1.

Additional File Table 2: Multivariate stepwise logistic regression analysis for predicting PCa in the training group for Model 2.

## Data Availability

The data are not publicly shared because we do not have permission from the Ethics Review Committee to distribute the data. The analytic methods are available from the corresponding authors upon reasonable request.

## Abbreviations

PCa: prostate cancer
DRE: digital rectal examinations
MRI: magnetic resonance imaging
TRUS: transrectal ultrasonography
PSA: prostate-specific antigen
PV: prostate volume
PSAD: prostate-specific antigen density
TC: cholesterol
TG: triglyceride
HDL: high-density lipoprotein
LDL: low-density lipoprotein
apolipoprotein AI: apo AI
apolipoprotein B: apo B
ROC: receiver operator characteristic
AUC: area under the curve
DCA: decision curve analysis
