# Supplementary material for: Establishment and validation of serum lipid-based nomogram for predicting the risk of prostate cancer
Source: BMC Urol. 2023 Jul 14;23:120. doi: 10.1186/s12894-023-01291-w (PMC10349516; doi:10.1186/s12894-023-01291-w)
Supplement: Supplementary file 1 — Additional File Table 1: Multivariate stepwise logistic regression analysis for predicting PCa in the training group for Model 1. [file 12894_2023_1291_MOESM1_ESM.docx]

**Supplementary Table 1** Multivariate stepwise logistic regression analysis for predicting PCa in the training group of Model 1.

| **Indicators** | **B** | **SE** | **P** | **Exp(B)** | **95% Exp(B)** |
| --- | --- | --- | --- | --- | --- |
| **PSA** | 1.178 | 0.241 | <0.001 | 3.248 | 2.026-5.206 |
| **f/t PSA** | -0.639 | 0.235 | 0.007 | 0.528 | 0.333-0.838 |
| **PSAD** | 0.599 | 0.237 | 0.012 | 1.820 | 1.144-2.897 |
| **DRE** | 0.786 | 0.256 | 0.002 | 2.195 | 1.330-3.624 |
| **TRUS** | 0.875 | 0.255 | 0.001 | 2.399 | 1.457-3.951 |
| **Constants** | -1.340 | 0.279 | <0.001 | 0.262 |  |

Model 1: Logit(PCa) = -1.340+1.178*PSA+-0.639*f/tPSA+0.599*PSAD+0.786*DRE(positive =1, negative=0)+0.875*TRUS(positive =1, negative=0)
