# Supplementary material for: Establishment and validation of serum lipid-based nomogram for predicting the risk of prostate cancer
Source: BMC Urol. 2023 Jul 14;23:120. doi: 10.1186/s12894-023-01291-w (PMC10349516; doi:10.1186/s12894-023-01291-w)
Supplement: Supplementary file 2 — Additional File Table 2: Multivariate stepwise logistic regression analysis for predicting PCa in the training group for Model 2. [file 12894_2023_1291_MOESM2_ESM.docx]

**Supplementary Table 2** Multivariate stepwise logistic regression analysis for predicting PCa in the training group of Model 2.

| **Indicators** | **B** | **SE** | **P** | **Exp(B)** | **95% Exp(B)** |
| --- | --- | --- | --- | --- | --- |
| **PSA** | 1.415 | 0.261 | <0.001 | 4.118 | 2.467-6.873 |
| **f/t PSA** | -0.623 | 0.248 | 0.012 | 0.536 | 0.330-0.871 |
| **PSAD** | 0.561 | 0.248 | 0.024 | 1.752 | 1.077-2.851 |
| **TG** | 0.681 | 0.299 | 0.023 | 1.976 | 1.100-3.548 |
| **LDL** | 1.471 | 0.302 | <0.001 | 4.353 | 2.408-7.868 |
| **DRE** | 0.889 | 0.269 | 0.001 | 2.432 | 1.436-4.119 |
| **TRUS** | 0.771 | 0.266 | 0.004 | 2.162 | 1.284-3.643 |
| **Constants** | -1.958 | 0.326 | <0.001 | 0.141 |  |

Model 2: Logit(PCa) = -1.958+1.415*PSA+-0.623*f/tPSA+0.561*PSAD+0.681*TG+1.471*LDL+0.889*DRE(positive =1, negative=0)+0.771*TRUS(positive =1, negative=0)
